# Transcriptome Comparative Profiling of Barley *eibi1* Mutant Reveals Pleiotropic Effects of *HvABCG31* Gene on Cuticle Biogenesis and Stress Responsive Pathways

**DOI:** 10.3390/ijms141020478

**Published:** 2013-10-14

**Authors:** Zujun Yang, Tao Zhang, Tao Lang, Guangrong Li, Guoxiong Chen, Eviatar Nevo

**Affiliations:** 1 School of Life Science and Technology, University of Electronic Science and Technology of China, Chengdu 610054, Sichuan, China; E-Mails: zhangtao@uestc.edu.cn (T.Z.); langtao123xxx@126.com (T.L.); ligr28@uestc.edu.cn (G.L.); 2 Extreme Stress Resistance and Biotechnology Laboratory, Cold and Arid Regions Environmental and Engineering Institute, Chinese Academy of Sciences, Lanzhou 730000, Gansu, China; E-Mail: guoxiong@hotmail.com; 3 Institute of Evolution, University of Haifa, Mount Carmel, Haifa 31905, Israel; E-Mail: nevo@research.haifa.ac.il

**Keywords:** Affymetrix GeneChip, barley, *eibi1*, *HvABCG31* mutant, transcriptome analysis

## Abstract

Wild barley *eibi1* mutant with *HvABCG31* gene mutation has low capacity to retain leaf water, a phenotype associated with reduced cutin deposition and a thin cuticle. To better understand how such a mutant plant survives, we performed a genome-wide gene expression analysis. The leaf transcriptomes between the near-isogenic lines *eibi1* and the wild type were compared using the 22-k Barley1 Affymetrix microarray. We found that the pleiotropic effect of the single gene *HvABCG31* mutation was linked to the co-regulation of metabolic processes and stress-related system. The cuticle development involved cytochrome P450 family members and fatty acid metabolism pathways were significantly up-regulated by the *HvABCG31* mutation, which might be anticipated to reduce the levels of cutin monomers or wax and display conspicuous cuticle defects. The candidate genes for responses to stress were induced by *eibi1* mutant through activating the jasmonate pathway. The down-regulation of co-expressed enzyme genes responsible for DNA methylation and histone deacetylation also suggested that *HvABCG31* mutation may affect the epigenetic regulation for barley development. Comparison of transcriptomic profiling of barley under biotic and abiotic stresses revealed that the functions of *HvABCG31* gene to high-water loss rate might be different from other osmotic stresses of gene mutations in barley. The transcriptional profiling of the *HvABCG31* mutation provided candidate genes for further investigation of the physiological and developmental changes caused by the mutant.

## 1. Introduction

The drought-hypersensitive mutant *eibi1* was obtained from a wild barley (*Hordeum spontaneum* Koch) accession in Israel [1]. The excessive water loss of the *eibi1* mutant plant was related to a recessive mutation localized in a pericentromeric region of chromosome 3H [2]. Studies also revealed severe effects on plant morphology, in particular, the reduced leaf cuticle development, which was associated to the high-water loss rate. Recently, a candidate gene for *eibi1*, based on high resolution genetic mapping was also reported [3]. A mutation on *HvABCG31*, an ATP-binding cassette (ABC) subfamily G (ABCG) full transporter, was associated with the *eibi1* phenotype [4].

The map-based cloning offers a promising relationship between candidate genes and a corresponding phenotypic trait [5]. However, the difficulty of barley transformation could not easily allow the functional analysis of the candidate gene with respect to its phenotypes. Recently, the rapid development of genomics based on high-throughput sequencing technologies, has facilitated the establishment of the function of target genes [6]. Availability of microarray platforms representing a large proportion of barley genes has enabled the application of transcriptomic analysis to several known mutations in barley including biotic and abiotic stress-related genes [7–11].

To better understand how *eibi1*/*HvABCG31* mutant displayed the defective physiological and growth phenotypes, we performed a genome-wide gene expression analysis by using Affymetrix Barley1 GeneChip microarray. We found that apparent compensatory transcriptional responses in the mutant involved metabolic processes and stress-related pathways. The comparative analysis of *eibi1* to other barley transcriptome components under various stress response signals was also revealed.

## 2. Results and Discussion

### 2.1. Differential Transcriptomes of *eibi1* Compared with the Wild Type

**Figure 1:**
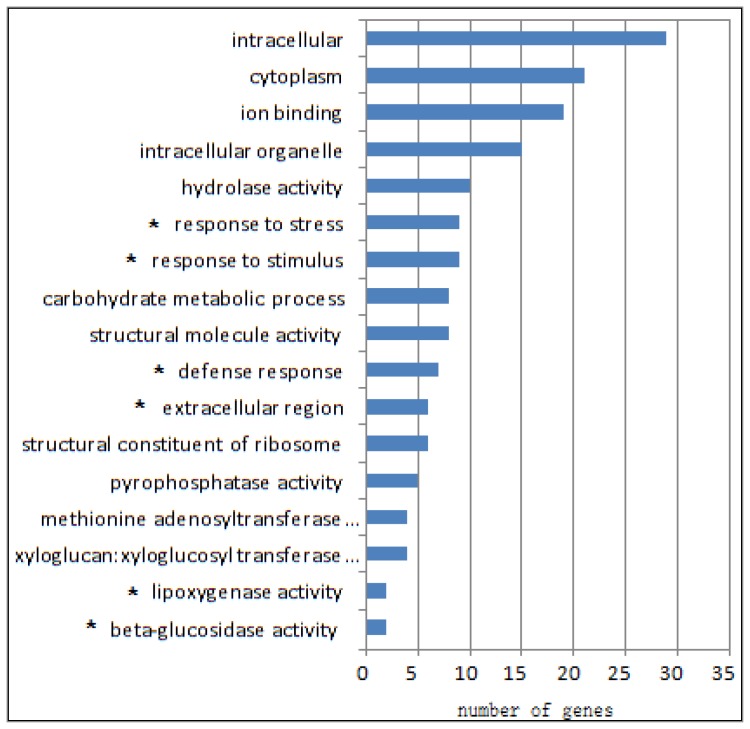
Differentially expressed genes in *eibi1* according to gene onthology. Stars indicate the up-regulated genes by *eibi1*, while others are down-regulated by *eibi1*.

To investigate the *eibi1* effects on downstream barley genes, we performed a microarray analysis using Affymetrix barley genome array chips. Out of 22,810 contigs on the chip, 488 (2.2%) contigs were up-regulated more than 2.0-fold, and 717 (3.7%) contigs were down-regulated to less than 0.5-fold in *eibi1* compared to the wild type (Figure 1).

The selected contigs from the Affymetrix genome array chip results involved in biological processes, cellular components, and molecular functions were analyzed by a GO term enrichment tool (Figure 1). Since several different contigs represent a single gene, a total of 164 genes were differentially (both up and down-regulated) expressed in *eibi1* compared with the wild type, involved in secondary metabolism, including cell skeleton, primary metabolism, signal transduction, cell growth and cell division, and defense responses. The secondary metabolism biosynthesis genes, some transcription factors and genes belonging to different functional categories were down-regulated in *eibi1* compared to the wild type. The genes involved in defense to stresses and lipid biosynthesis were up-regulated in *eibi1* compared to the wild type.

Moreover, we also observed that a total of eight contigs involved in transport, including sugar transporters (Contig6706_at, HY05O16u_s_at) and iron transporters (HV_CEa0013E09r2_at, Contig6152_at) were up-regulated significantly in *eibi1* compared to wild type. Similarly, six genes involved in transport, including two ABC transporters (Contig6016_s_at, Contig5296_at) and CorA-like Mg^2+^ transporters (Contig10637_s_at, Contig10636_at), were down-regulated in *eibi1* compared to the wild type. The *eibi1* mutant was caused by ABC transporter *HvABCG31* gene mutation. The transcriptome profiling indicated that the *HvABCG31* was not differentially expressed in leaves. Therefore, effect of *eibi1* mutant on downstream genes was associated the defect of leaf phenotype.

### 2.2. Stress and Fatty Acid Metabolism Related Pathways Were Up-Regulated

As shown in Table 1, we found that four contigs (Contig1579_s_at, Contig1570_s_at, Contig1582_x_at, Contig1580_x_at) were up-regulated by 4–39-fold, as were all encoded Thionins, which are low-molecular-weight proteins (Mr ca. 5 kD) occurring in seeds, stems, roots, and leaves of a number of plant species. The different members of this plant protein family show both sequence and structural homology, and are toxic to bacteria, fungi, yeasts, and various naked cells *in vitro* [12].

The Contig 6157_s_at, representing Horcolin (*Hordeum vulgare* coleoptile lectin), was up-regulated by 9.402-fold in *eibi1*. Database searches performed with the Horcolin protein sequence revealed its structure homology to the lectin family of jacalin-related lectins (JRL). As a new member of the mannose-specific subgroup of jacalin-related lectins in monocot species [13], overexpression of a wheat jasmonate-regulated lectin increases pathogen resistance, and the group of inducible lectins appears to function within the context of biotic/abiotic stress signaling in monocots and dicots [14,15]. Defense-related genes were up-regulated in the *eibi1* leaves including ribosome inactivating proteins, chitinases (Contig2990_at), protease inhibitors, amylases (Contig1411_s_at) and glucanases (HVSMEm0003C15r2_s_at). The function of the chitinases and b-glucanases may degrade the major structural component of the cell walls of many fungi [16].

The contigs for the fatty acid metabolism pathway were up-regulated, such as Contig5664 encoding very-long-chain fatty acid condensing enzyme, and HVSMEn0002B08r2_at encoding fatty acid elongase were up-regulated in *eibi1* compared to the wild type. The enzymes called lipoxygenases (LOXs) (Contig 12574_at, Contig23795_at, Contig2305_at) can dioxygenate unsaturated fatty acids, which leads to lipoperoxidation of biological membranes. LOXs are known to be involved in the apoptotic (programmed cell death) pathway, and biotic and abiotic stress responses in plants [17]. GDSL-motif lipase/hydrolase family protein was reported to have protein localization and gene expression patterns that correlated with cutin biosynthesis [18] and the abundance of transcripts for GDSL-motif lipase/hydrolase, thought to contribute to cuticle reorganization and increased permeability [19]. The Contig15_s_at representing GDSL-motif lipase/hydrolase was significantly up-regulated 6-fold in *eibi1* compared to the wild type.

Since the common activation of the stress-related genes of Thinion, Horcolin, and other lipid synthesis enzymes, their transcription was shown to be methyl jasmonate (MeJA)-inducible in leaves [20]. A total of eight out of 33 contigs involving methyl jasmonate-inducible protein were up-regulated; none of the contigs involved in Methyl jasmonate-inducible protein were down-regulated in the present differential analysis. The result suggested that *eibi1* mutation can possibly activate the MeJA-inducible pathway in the response to stresses. Moreover, it is interesting to note that Horcolin was only expressed in barley coleoptiles [21]; however, it is highly expressed in leaves of *eibi1*. Considering the *eibi1* candidate gene, ABC transporter was also confined to the coleoptiles, we hypothesized that the mutation of *eibi1* may clearly affect the expression of horcolin, which may mediate related pathways for the response to stresses. We also found that by microarray gene expression profiling, *eibi1* was similar to the reports of mutants *lcr*, *fdh*, and *bdg,* indicating that a number of up-regulated defense-related genes had accumulated [22]. The overall activation of a number of stress responsive genes in *eibi1* may be associated with high resistance to barley fungi including rust and powdery mildew (data not shown), although *eibi1* plant appeared thin leaves.

Cytochrome P450 monooxygenases (CYP450) catalyze substrate-, region- and stereo-specific oxygenation steps in plant metabolism. Co-expression results for CYP450 related to plastidial functions/photosynthesis, and to phenylpropanoid, triterpenoid, and jasmonate metabolism [23]. In the present study, we found that eight contigs (Contig11818_at, Contig25477_at, Contig3160_at, Contig13847_at, Contig14804_at, Contig7194_at, Contig14663_at, and ContigCEb0006F_at) were up-regulated significantly in *eibi1* compared to wild type. None of the contigs homologous to CYP450 were down-regulated in *eibi1* compared to WT. Contig25477_at was up-regulated by 3.52-fold in *eibi1*, and it was homologous to At4g39490, a paralog of At1G57750 (AtMAH1) protein [24], which was involved in the cutin monomer synthesis [25]. It might be interpreted that cuticular mutant *eibi1* would up-regulate the CYP450 related pathway to reduce levels of cutin monomers or wax and display conspicuous cuticle defects. Notably, the CYP450 family can be a candidate to investigate the mechanism of cutin synthetic pathway in *eibi1*.

### 2.3. The Metabolic Pathways Were Down-Regulated

We found that all the Contigs (Contig2670_x_at; Contig2670_s_at and Contig2672_at) encoding xyloglucan transglycosylases (XETs) were clearly down-regulated 3-fold in *eibi1* (Table 2). The XETs have been implicated in many aspects of cell wall biosynthesis [26]. The Contig5258_at encoding Endoxyloglucan transferase (EXT) was down-regulated 2.5-fold in *eibi1*. Xyloglucans are the principal components of the matrix polymers and bind tightly to the surface of cellulose microfibrils and, thereby, cross-link them to form an interwoven xyloglucan-cellulose network structure [27]. EXT is a newly identified class of transferase that catalyzes molecular grafting between xyloglucan molecules, thereby mediating molecular grafting between xyloglucan cross-links in plant cell walls [28]. The down-regulated of XETs and EXT genes in *eibi1* may possibly affect the cell shapes and resulted in thin leaves.

The UDP-xylose is an important sugar donor in the synthesis of hemicellulose and glycoproteins [29]. Arabinoxylans in crop plants such as rice, maize, wheat, and barley are major components of the cell wall of the starchy endosperm, as well as the aleurone layer [30]. UDP-d-glucuronic acid decarboxylase (UXS) (Contig2915_at 0.409, Contig2031_at 0.206) catalyzed the conversion of UDP-d-glucuronic acid to UDP-d-xylose. UDP-xylose is not only used as a substrate for xyloglucan biosynthesis, but also as a substrate of β-(1,4)-xylosyltransferase that catalyses the synthesis of the xylan backbone [31]. The down-regulated of these genes may be associated with the low plant height and grain traits of *eibi1* compared with the wild type.

Actin, which is vital for pectin synthesis and for cytoskeleton [32], is significantly down-regulated (Contig1390_M_at) 7-fold in the *eibi1* mutant. Phenylalanine ammonia-lyase (PAL) (Contig1805_s_at, Contig1803 _at, Contig1800_at, HVSMEm0015M15r2_s_at) was down-regulated (Table 2). It catalyzes the first reaction in the general phenylpropanoid pathway leading to the production of phenolic compounds with a significant range of biological functions. The heat shock protein members HSP80 and HSP90 were down-regulated 2.1–2.3-fold in the *eibi1* mutant. In the case of inactivation of heat-shock protein synthesis, it is well recognized the damaged/denatured proteins signals in *eibi1*.

The transcriptome profiling of molecular pathways in *eibi1* were down-regulated, in particular, the genes involved in cell wall modification. The expression of the related gene network underlying cell wall biosynthesis will deteriorate the cuticle development, particularly as is evident in the thinner leaves and shorter plants of *eibi1* than that of wild type.

### 2.4. The Epigenetic Related Pathways

Epigenetic phenomena have been associated with the regulation of active and silent chromatin states achieved by modifications of chromatin structure through DNA methylation and histone post-translational modifications [32].

As shown in Table 3, the micro-molecular proteins involved in methionine synthase (MSY) (Contig1424_at) were down-regulated 4-fold in *eibi1*. All three enzymes, MSY, SAM1 (*S*-adenosyl-l-methionine synthetase), and SAH (*S*-adenosyl-l-homocysteine hydrolase) were involved in the methyl cycle [33]. The co-expressed enzyme genes are likely responsible for DNA methylation involved in determining plastid division and amyloplast differentiation as included in seeds development of barley [34].

Expression of *S*-Adenosylmethionine Synthetase gene (SAM1) appeared to be 2–3-fold down-regulated (Contig1269_at, Contig1269_s_at and Contig1271) in *eibi1* compared to WT (Table 3). SAM catalyzes the biosynthesis of *S*-adenosyl-l-methionine (AdoMet), a universal methyl-group donor. In *Arabidopsis* and rice, the SAM gene is expressed primarily in the vascular tissue and is also preferentially accumulated in lignified tissues [35]. Moreover, we found that SAH (Contig1791_x_at and Contig1794_at) were clearly down-regulated by 2–5-fold in *eibi1* compared to WT (Table 3). The *S*-adenosyl-l-homocysteine metabolic pools were highly regulated on cytosine methylation [36], and down-regulation of SAH reveals the role of cytokinin in promoting transmethylation reactions [37].

Histon deacetylases (HDAC) are important in plant gene expression (Contig1625_at). HDAC function has been best studied in *Arabidopsis*. Inactivation of *Arabidopsis* HD1 (AtHD1/HDA19), mutant (athd1-t1) is induced by pleiotropic developmental abnormalities [38,39]. Down-regulation of HDAC reduced rice peduncle elongation and fertility, altered plant height and flag leaf morphology, leading to the production of narrowed leaves and stems [40].

The co-expression of the contigs involved in the methylation and histon modification in *eibi1* and WT infer that the *eibi1* may affect the epigenetic pathways. The relationship of phenotypical changes between *eibi1* and the WT with respect to the epigenetic modification need to be further investigated.

### 2.5. Validation of Microarray Data by qRT-PCR

To assess the accuracy of microarray data, we selected 10 differential expressed contigs including stress responsive genes, secondary metabolism biosynthesis genes and epigenetic modifying genes as shown in Table 4. The expression profiles of the genes that show up or down-regulated up-regulation between *eibi1* and WT identified by microarray. We tested the similarity between gene expression identified by microarray and those by qRT-PCR, and observed that microarray and qRT-PCR data, which were calculated based on the median of three repeats, showed good correlation at different water stress treatments and overall water stress conditions (*r* = 0.902–0.960). Hence, the results of the differential expressed genes identified through microarrays confirm actual differences between *eibi1* and WT genotypes.

### 2.6. Transcriptomic Comparison of *eibi1* with Other Barleys under Stresses

**Figure 2:**
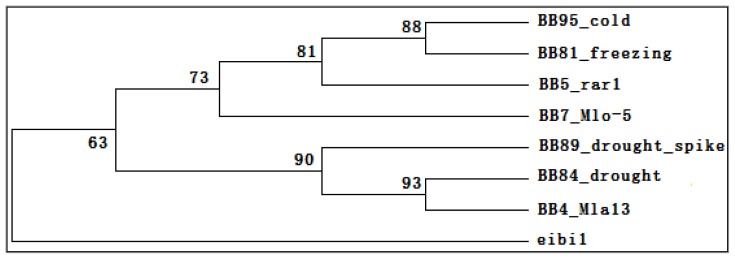
Hierarchical cluster analysis of differentially expressed genes from *eibi1* and barley cultivars transcriptome change under various stress treatments (from data available at PlexDB database).

Since the whole genome transcript analysis revealed that *eibi1* activated some signaling pathways in response to stress factors, we performed a comparative analysis of differentially expressed genes from *eibi1* to other barleys’ transcriptome change under various stress treatments on PlexDB database [41–43]. The available Affymetrix Barley1 GeneChip data included barley transcriptome change in response to four abiotic factors including chilling and freezing temperature (BB81, BB95), drought (BB84, BB89), and three biotic factors from powdery mildew resistance genes mlo-5 (BB7), mla-13 (BB4) and rar1 (BB5). The differentially expressed genes from *eibi1* down-regulated more than 2-fold as compared to WT were selected for the cluster analysis (Figure 2). The cluster analysis showed that the set of differentially expressed genes from *eibi1* was different from the data from all analyzed datasets, and was clustered as an out group.

Based on the cluster analysis, we supposed that the *eibi1* mutant might have different reaction mechanisms for the osmotic stress of high water loss rate, which is not similar to the stress-induced expressional changes and other reported mutations for barley biotic and abiotic stresses.

## 3. Experimental

### 3.1. Plant Materials and Experimental Design

**Figure 3:**
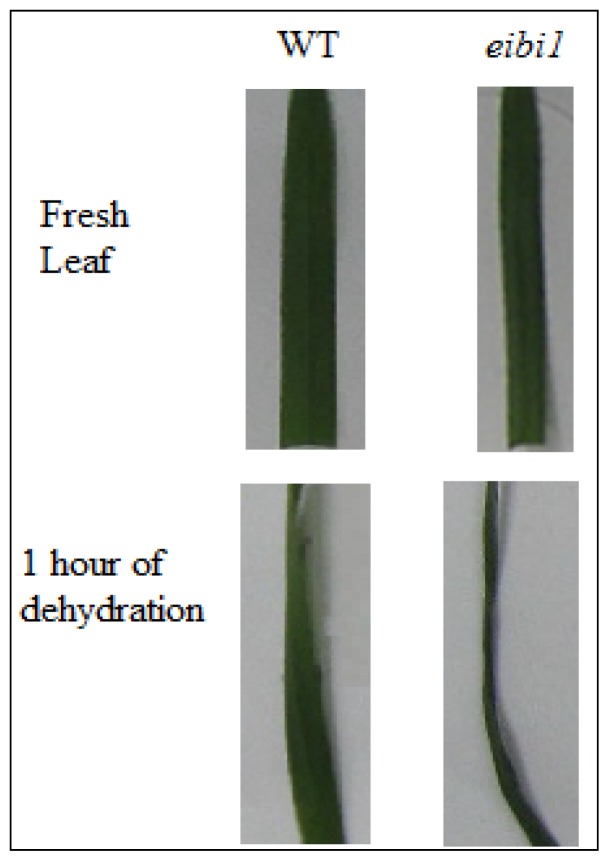
Phenotypes of leaves of eibi1 and wild type before or after dehydration.

Germplasm used for this study was derived from *eibi1* mapping population [2]. The F_3_ populations of 23–19/eibi1 for BSA analysis were chosen to evaluate the water loss rate [3]. We isolated the second leaves of the seedling after 10 days after germination at 22 °C in growth chamber. The detached leaves of *eibi1* plants lost about 50% of their initial weight during 1 h of dehydration while the wild type detached leaves lost only 5% (Figure 3). Half leaves were immediately frozen by liquid nitrogen, and the other half was used to test the water loss rate. Each of the 20 leaves with significant water loss rate representing the genotype of *eibi1* and normal water loss rate for wild type was mixed, respectively, and three replicates of samples were used for RNA extraction and microarray analyses.

### 3.2. Leaf Transcriptomic Microarray Analysis

Total RNAs were extracted from each replicate using the RNeasy Mini Kit (Qiagen). Each sample was hybridized to a barley chip (22-k Barley1 DNA microarray, Affymetrix, Santa Clara, CA, USA). Chip preparation, hybridization and analysis were done at the CaptitalBio Corporation (Beijing, China). cDNA synthesis (first and second strand), biotin-labeled cRNA synthesis, fragmentation, hybridization, washing, staining, and scanning were performed according to the standard manufacturer’s (Affymetrix) recommendations. The microarray data were further analyzed using the GOEAST program [44].

### 3.3. Quantitative RT-PCR Validation of *Eibi1* and Wild Type Gene Expression

Microarray data were further validated using qRT-PCR for a selected number of genes using gene-specific primer sets. Primer pairs (Table 4) were designed using OligoPerfect Designer software [45]. Specificity of the primer sets and their product length was verified by agarose gel electrophoresis. The qRT-PCR reaction mixture was consist of the SYBR Premix EX-Taq™ II Kit (TakaRa, Japan) on iCycler iQ (BIO-RAD) with three biological repeats of cDNA of *eibi1* and wild type.

### 3.4. Comparative Transcriptomic Analysis

The differentially expressed (at least two-fold up or down-regulated) probe sets from our experiment were compared with the expression of the same probe sets in public barley GeneChip experiments from the PlexDB database [43]. Seven transcriptome changes representing barley in response to abiotic and biotic factors were chosen. Bootstrap confidence levels of transcriptome analysis of the barley *eibi1* mutant were calculated from 100 iterations using the seqboot programme from the PHYLIP package. A graphical tree representing the comparison was visualized using TreeView [46].

## 4. Conclusions

The phenotypic observation of leaf profiles suggested that *eibi1* appeared significant effects on the cuticle formation compared to the wild type. We hypothesize that the differences in *eibi1* leaf cells might not be under the direct control of the *HvABCG31* gene, but are probably the result of pleiotropic effects of the mutation. Based on the present whole genome expression analyses on *eibi1* leaves compared with wild-type leaves, we found that the pleiotropic effects of *eibi1* mutation were primarily activated by CYP450 regulated genes, fatty acid metabolic pathway, and the jasmonate signal transduction pathway, as well as, possibly, epigenetic related pathways to deal with the osmotic stress of high water loss rate in leaves. We conclude that the single gene mutation of *eibi1* showed that the unique *in vivo* dehydration stress leading to the morphological and physiological changes, and the genetic and epigenetic mechanisms responsible for the *eibi1* plant defects were different from the reported barley abiotic mutation or treatments.

## Figures and Tables

**Table 1:** The up-regulated stress related marker/gene in *eibi1*.

(Putative) gene function	Contig/gene designation	Regulation in *eibi1*/WT
Thionin	Contig1579_s_at	39.5
	Contig1570_s_at	18.5
	Contig1582_x_at	6.96
	Contig1580_x_at	4.07
Horcolin	Contig6157_s_at	9.42
Pathogen-related protein pir	Contig5607_s_at	4.87
Methyl jasmonate-inducible lipoxygenase 2	Contig2305_at	2.059
Lipoxygenase	Contig23795_at	3.729
Metallophosphatase	Contig2289_at	2.409
carbonate dehydratase	Contig897_s_at	2.363
Chitinase	Contig2990_at	5.87
β-amylase	Contig1411_s_at	5.03
RNase S-like protein	contig5059_s_at	2.212
Lectin	contig11641_s_at	2.099
subtilisin-like protease	Contig13847_s_at	3.188
α-L-arabinofuranosidase/β-D-xylosidase	Contig7032_s_at	2.842
endo-1,3-β-glucanase	HVSMEm0003C15r2_s_at	2.609
glutathione synthetase	Contig14516_at	2.379
GDSL-motif lipase/hydrolase	Contig15_s_at	6.113
CYP450		
	Contig 11818_at	5.409
	Contig 25477_at	3.52
	Contig3160_at	3.235
	Contig13847_at	3.191
	Contig14804_at	2.308
	Contig7194_at	2.292
	Contig14663_at	2.111
	ContigCEb0006F_at	2.018
Fatty acid	Contig23795_at	3.729
	Contig5664_at	2.535
	HVSMEn0002B08r2_at	2.164
	Contig2305_at	2.059

Differential gene regulation in 2nd leaves of *eibi1* compared to WT based on microarray analysis. Genes up regulated at >2-fold.

**Table 2:** Differentially down-regulated genes in *eibi1*.

(Putative) gene function	Contig/gene designation	Regulation in *eibi1*/WT
ribophorin I	Contig4748_s_at	0.499
sucrose synthase 1	Contig689_s_at	0.499
dTDP-glucose 4-6-dehydratase-like protein	Contig2918_s_at	0.48
UDP-glucuronic acid decarboxylase	Contig2915_at	0.469
UDP-glucuronic acid decarboxylase	Contig2031_s_at	0.206
ribosomal protein S8	Contig1024_at	0.467
60S	Contig2369_s_at	0.43
ribosomal protein L24	HI02E24u_s_at	0.331
rpS28	Contig3403_s_at	0.321
ribosomal protein L17.1, cytosolic	rbags1i23_s_at	0.302
Ribosomal protein S7	Contig1668_at	0.273
peroxidase (EC 1.11.1.7), pathogen-induced	Contig2118_at	0.325
HSP80-2	Contig1204_s_at	0.433
Endoxyloglucan transferase (EXT)	Contig5258_at	0.423
Serine carboxypeptidase III precursor (CP-MIII)	Contig682_s_at	0.413
glutathione peroxidase-like protein	Contig2453_at	0.406
Pyrophosphate phospho-hydrolase (PPase)	Contig2018_at	0.389
phenylalanine ammonia-lyase	Contig1805_s_at	0.496
phenylalanine ammonia-lyase (EC 4.3.1.5)	Contig1803_at	0.37
phenylalanine ammonia-lyase (EC 4.3.1.5	HVSMEm0015M15r2_s_at	0.366
phenylalanine ammonia-lyase (EC 4.3.1.5)	Contig1800_at	0.353
Glyceraldehyde 3-phosphate dehydrogenase	Contig149_at	0.361
immunophilin	HVSMEg0002K18r2_s_at	0.358
ascorbate peroxidase	Contig1727_s_at	0.242
plasma membrane proton ATPase	Contig2_s_at	0.296
xyloglucan endotransglycosylase (XET)	Contig2670_x_at	0.3
xyloglucan endo-1,4-β-D-glucanase(XET)	Contig2672_at	0.335
xyloglucan endo-1,4-β-D-glucanase	Contig2670_s_at	0.358
promoter-binding factor-like protein	Contig10705_at	0.443
Actin	Contig1390_M_at	0.187
serine carboxypeptidase III	Contig682_s_at	0.413
glutathione peroxidase-like protein GPX54Hv	Contig2453_at	0.406

Differential gene regulation in second leaves of *eibi1* compared to WT based on microarray analysis. Genes were down-regulated at >2-fold.

**Table 3:** Differentially expressed epigenetic modifying genes.

(Putative) gene function	Contig/gene designation	Regulation in *eibi1*/WT
Methionine adenosyltransferase 1	Contig1269_at	0.456
Methionine adenosyltransferase 1	Contig1271_x_at	0.427
Methionine adenosyltransferase 1	Contig1269_s_at	0.327
*S*-adenosyl-L-homocysteine hydrolase	Contig1791_x_at	0.477
*S*-adenosyl-L-homocysteine hydrolase	Contig1794_s_at	0.271
methionine synthase protein	Contig1424_at	0.244
putative histone deacetylase HD2	Contig1625_at	0.348

Differential gene regulation in second leaves of *eibi1* compared to WT based on microarray analysis. Genes were down-regulated at >2-fold.

**Table 4:** The PCR primers for selected genes.

Contig	Predict functional gene	EST accession	Primers
Contig1579_s_at	Thionin	AK250720	TGAATCTTCTCCCTGAATCCG CAAATAGATTCATCGTGGCGA
Contig6157_s_at	horcolin	AY033628	CTACGTGACCGAAATCTCCG GCCATGTAAGGCACTTCACAA
Contig2990_at	chitinase	X78671	CAACACCTTTCCGGGCTT CCGTCATCCAGAACCACATC
Contig 25477_at	CYP450	AK252707	CTTCCACAACGACCCTGACT AGCACCTGCACATCAAAGTTC
Contig23795_at	lipoxygenase	AJ507213	TGATCCATCTGAAGCAGCCT TTGACGGTGAAGAAGGCG
Contig23796_at	Fatty acid	AJ507214	CGCAGAGCAGCAATGTTG GCGACGGCAGGTATGACTT
Contig2031_s_at	UDP-glucuronic acid decarboxylase	AY677177	TGAATCTTCTCCCTGAATCCG CAAATAGATTCATCGTGGCGA
Contig1269_s_at	Methionine adenosyltransferase 1	AK249878	CTACGTGACCGAAATCTCCG GCCATGTAAGGCACTTCACAA
Contig1794_s_at	*S*-adenosyl-L-homocysteine hydrolase	AM039898	CAACACCTTTCCGGGCTT CCGTCATCCAGAACCACATC
Contig1424_at	methionine synthase protein	AM039905	CTTCCACAACGACCCTGACT AGCACCTGCACATCAAAGTTC
